# Transcription factors and stress response gene alterations in human keratinocytes following Solar Simulated Ultra Violet Radiation

**DOI:** 10.1038/s41598-017-13765-7

**Published:** 2017-10-19

**Authors:** Thomas L. Des Marais, Thomas Kluz, Dazhong Xu, Xiaoru Zhang, Lisa Gesumaria, Mary S. Matsui, Max Costa, Hong Sun

**Affiliations:** 1 New York University, Department of Environmental Medicine, Tuxedo, New York, United States of America; 2 Estee Lauder Companies, Inc., Melville, New York, United States of America; 3 New York Medical College School of Medicine, Department of Pathology, Valhalla, New York, United States of America

## Abstract

Ultraviolet radiation (UVR) from sunlight is the major effector for skin aging and carcinogenesis. However, genes and pathways altered by solar-simulated UVR (ssUVR), a mixture of UVA and UVB, are not well characterized. Here we report global changes in gene expression as well as associated pathways and upstream transcription factors in human keratinocytes exposed to ssUVR. Human HaCaT keratinocytes were exposed to either a single dose or 5 repetitive doses of ssUVR. Comprehensive analyses of gene expression profiles as well as functional annotation were performed at 24 hours post irradiation. Our results revealed that ssUVR modulated genes with diverse cellular functions changed in a dose-dependent manner. Gene expression in cells exposed to a single dose of ssUVR differed significantly from those that underwent repetitive exposures. While single ssUVR caused a significant inhibition in genes involved in cell cycle progression, especially G2/M checkpoint and mitotic regulation, repetitive ssUVR led to extensive changes in genes related to cell signaling and metabolism. We have also identified a panel of ssUVR target genes that exhibited persistent changes in gene expression even at 1 week after irradiation. These results revealed a complex network of transcriptional regulators and pathways that orchestrate the cellular response to ssUVR.

## Introduction

Solar ultraviolet radiation (UVR) is one of the most impactful environmental factors affecting human skin and leads to an array of photo damage endpoints, including sunburn, erythema, edema, immune suppression, as well as skin cancers^1^. Epidemiological studies have established an association between chronic or intense sun exposure and increased risk of melanoma and non-melanoma skin cancers^2^. The ozone layer effectively blocks most solar radiation that has wavelength less than 290 nm, so that the UVR reaching the skin surface is a mixture of 5–10% UVB (290–320 nm) and 90–95% of UVA (320–400 nm). Both UVA and UVB are able to induce DNA damage in mammalian cells. Exposure to UVB directly targets DNA and induces two major types of DNA lesions: cyclobutane pyrimidine dimers (CPDs) and (6–4) pyrimidine-pyrimidone photoproducts [(6–4)PPs]. UVA induces reactive oxygen species (ROS) that generate oxidative DNA damage (such as 8-oxo-deoxyguanine)^3,4^. Failure to repair these DNA lesions can result in either cell death or an accumulation of mutations, which in turn may lead to skin tumor initiation and progression.

The mammalian skin is the largest organ that covers the surface and protects the body from various external stimuli including solar UV radiation^5^. Skin consists of two main layers: the epidermis that is composed of keratinocytes, melanocytes and Langerhans cells, and the dermis that contains fibroblasts, fibrocytes, nerve ending, vasculature and immune cells^5^. Besides its function as a protective barrier, skin produces cytokines, neurotransmitters and hormones, which actively regulate both local and global homeostasis through its melatoninergic and steroidogenic system^5–7^. Recent studies showed that keratinocytes produce melatonin and its metabolites as well as several novel vitamin D3 metabolites to protect skin cells against UVR-induced damage^7–9^.

Moreover, cells have developed a highly-sophisticated response (DNA damage response, DDR) to protect the integrity of the genome. Multiple DNA repair pathways are actively involved in repairing UVR-induced DNA damage^3,4^. Simultaneously, DNA-damage induced signaling cascades are activated; DNA replication and transcription are temporarily stalled at the sites of damage; and chromosome segregation is paused at cell cycle checkpoints. These events lead to dynamic transcriptional changes that subsequently contribute to a series of divergent cellular UV responses, including DNA damage repair, cell cycle arrest, and apoptosis^10^.

Transcriptional changes induced by UV radiation have been extensively studied in skin cells both *in vitro* and *in vivo*, including keratinocytes, fibroblasts, melanocytes, and human epidermis *in vivo* ^11–19^. It should be noted that most of the studies have focused on UVR at a specific region of the spectrum such as UVA, UVB or UVC. Gene expression changes in response to solar UV radiation with a solar-simulated spectrum has not been fully characterized, particularly using repetitive irradiation protocols. A study on human monocyte-derived dendritic cells demonstrated that solar simulated UV radiation (92.5% UVA + 7.5% UVB) upregulated genes involved in cellular stress and inflammation and downregulated genes involved in chemotaxis, vesicular transport and RNA processing^20^. Recent studies on reconstructed skin indicated that daily UV radiation (DUVR, a mixture of 96.5% UVA and 3.5% UVB) modulated genes involved in cutaneous biology and stress response^21,22^. Using reconstructed skin, the expression of 225 and 241 genes were analyzed by quantitative PCR array in fibroblasts and keratinocytes respectively, which were exposed to either DUVR or UVA alone. Interestingly, while 97% of gene changes in fibroblast are modulated by both DUVR and UVA, only 80% of altered genes are regulated in common with keratinocytes, suggesting that dermal fibroblasts and epidermal keratinocytes have different responses after exposure to DUVR or UVA^21–23^. However, only a limited number of genes were analyzed in these studies.

We previously investigated global changes of histone post-translational modifications in human keratinocytes exposed to solar simulated UVR (ssUVR composing 95% UVA and 5% UVB)^24^. The acetylation of several lysine residues located on histone H3 and H4 was reduced after either a single dose of radiation or 3–5 repetitive exposure of ssUVR. Since acetylation neutralizes the positive charge of lysine residues and facilitates chromatin remodeling toward a more relaxed, open conformation, histone acetylation is normally associated with active gene expression^25,26^. To investigate whether ssUVR-induced hypoacetylation leads to changes in gene expression, we analyzed the expression profiles of human keratinocytes exposed to single or repetitive irradiation of ssUV. Differentially expressed genes following ssUVR were identified and subsequently analyzed for pathways and upstream regulators that may contribute to the cellular response to ssUV radiation.

## Results

### Transcriptional profiling of gene expression of HaCaT cells in response to ssUVR

**Figure 1:**
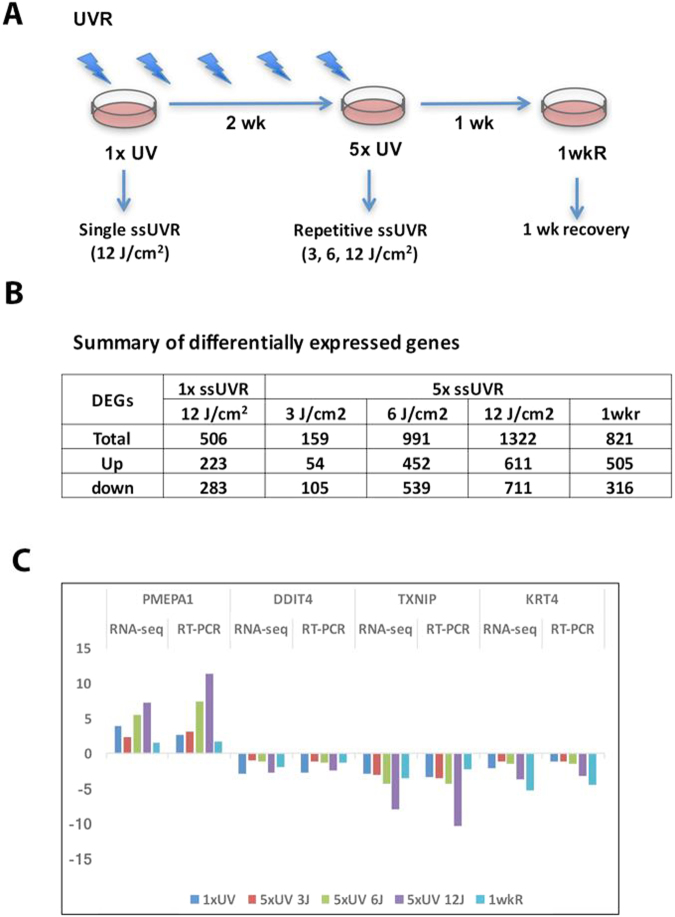
ssUV radiation modulates gene expression in HaCaT cells. (**A**) Schematic diagram showing the ssUV radiation and recovery of HaCaT cells. (**B**) Summary of differentially expressed genes in cells exposed to a single dose of radiation (1xssUVR), 5 repetitive radiation (5xssUVR), and 1 week recovery (1wkR) after repetitive radiation. (**C**) Validation of RNA-Seq results by quantitative real-time PCR. The PCR results are presented as fold change to the level expressed in untreated sham samples, and compared to those obtained from RNA-Seq.

Immortalized human keratinocyte HaCaT cells were exposed to either a single, high dose of ssUVR (12 J/cm^2^) or 5 repetitive doses of ssUVR (once every three days) at 3, 6 or 12 J/cm^2^ (Fig. 1A). The range of doses used in the study is comparable to what a person would be exposed to when standing outside for 40 minutes (3 J/cm^2^) to 2.5 hours (12 J/cm^2^) at noon under a clear sky with a UV index of 6 or higher^24^. Our previous study found that HaCaT cells exposed to a single dose of 12 J/cm^2^ exhibited a moderate increase of phosphorylated histone variant H2AX, a well-characterized marker of DNA damage, at 1 hour post irradiation, as well as limited cell death (less than 20%) at 24 hours after irradiation^24^. Irradiated cells were harvested 24 hours post irradiation and subjected to transcriptional profiling. To mimic human skin repetitively exposed to ssUVR, cells were exposed to 5 repetitive doses of ssUVR at 3, 6, 12 J/cm^2^ and were collected at 24 hours post irradiation. To examine the persistence of gene expression changes, cells were exposed to 5 repetitive doses of ssUVR at 12 J/cm^2^, and were collected at 1 week after the last irradiation. Two biological replicates of controls (sham) or irradiated cells (UVR) were collected for each dose or time point.

Characterization of gene expression changes induced by ssUVR was carried out by RNA-seq. Using the Illumina HiSeq. 2500 platform, a total of 0.6 billion reads were obtained from 16 RNA samples with an average of 38.2 million reads per sample. More than 90% of reads were mapped to human genome (Hg38). Data analysis was performed using Biomedical Genomics Workbench version 3.5.3 (Qiagen). Differential gene expression was analyzed using Advance RNA-seq plug-in tool (Qiagen) by comparing each treated group versus corresponding control group. The genes with a false discovery rate (FDR) < 0.05 between control and treated group and an average mean of total counts no less than 10 were defined as differentially expressed genes (DEGs). A total of 5 DEG lists were generated to represent genes changed in the different treatments (Supplemental Table 1). The numbers and the direction of regulation (Up or down) of DEGs in different exposure groups are summarized in Fig. 1B. In cells exposed to a single ssUVR at 12 J/cm^2^, a total of 506 DEGs genes were identified, of which 223 genes were up-regulated and 283 down-regulated. In cells exposed to 5 repetitive doses of ssUVR at 12 J/cm^2^, the total number of DEGs was increased to 1322, including 611 up-regulated and 711 down-regulated genes. In contrast, cells collected at 1 week post irradiation exhibited a reduced number of DEGs (n = 821) compared to those at 24 hours post irradiation. The number of DEGs was positively correlated with the exposure frequency of ssUVR, but negatively correlated with the recovery time. Moreover, classification of the protein functions of DEGs using Ingenuity Pathway Analysis (IPA) revealed that enzymes, transcriptional regulators, kinases, and transporters are the top represented categories in all five DEG lists (Supplemental Fig. 1).

In order to validate RNA-seq results, 4 genes were selected (PMEPA1, DDIT4, TXNIP, KRT4) based on their expression changes. While PMEPA1 is the top upregulated gene in cells exposed to either a single dose ssUVR or 5 repetitive ssUVR, DDIT4, TXNIP and KRT4 are among top 10 downregulated genes in both treatments (Supplemental Table 1). As shown in Fig. 1C, although the fold change of each gene from RT-qPCR is slightly different to those obtained from RNA-seq, the trend of gene regulation remains the same. Thus, the RNA-seq results indicate that ssUVR alters the global gene expression profile.

### Functional analysis of DEGs in HaCaT cells exposed to single ssUVR

**Figure 2:**
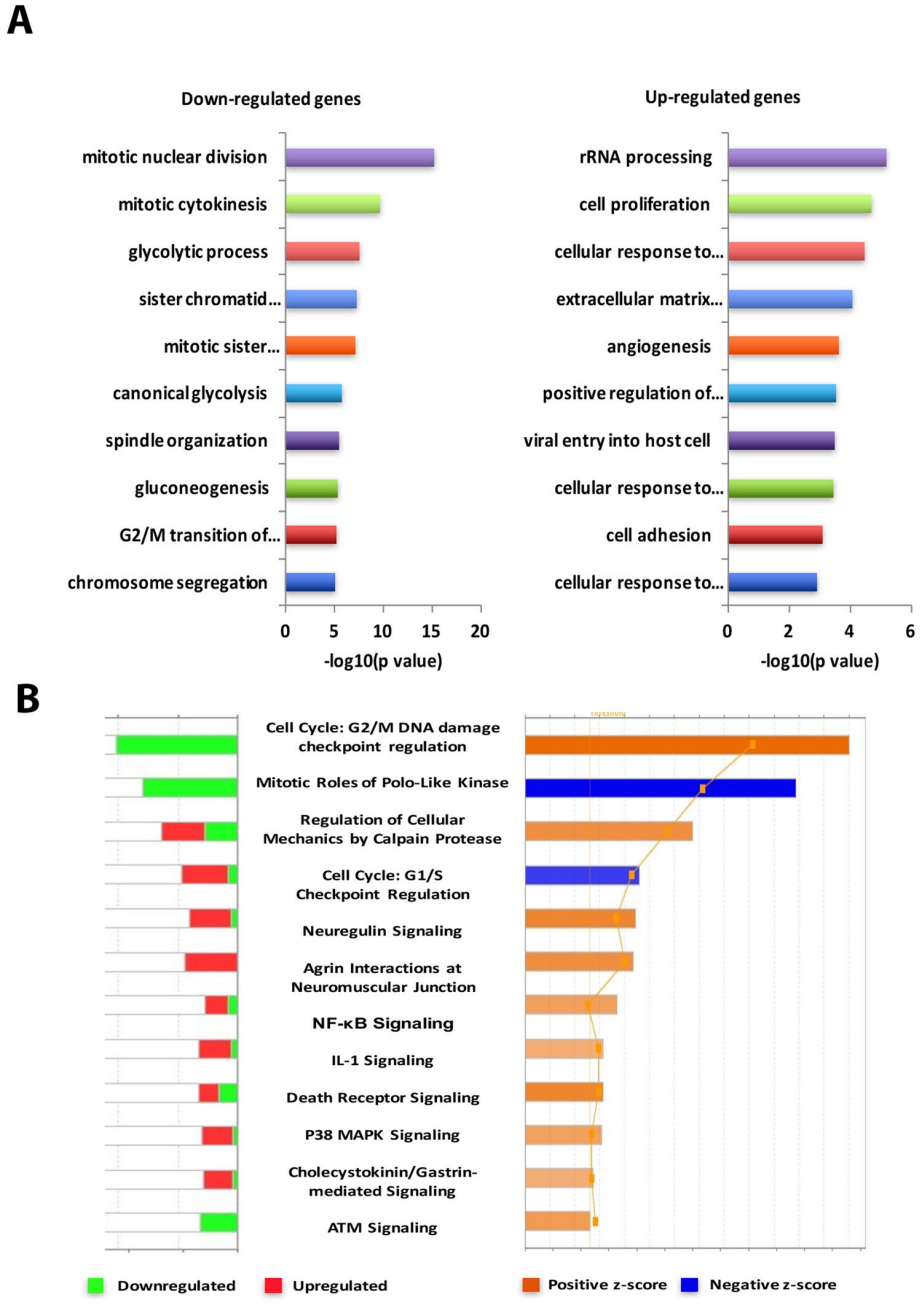
Functional annotation and pathway analysis of DEGs in HaCaT cells exposed to single ssUV radiation at 12 J/cm^2^. (**A**) The top biological process GO terms of down- regulated (left panel) and up-regulated (right panel) DEGs are ranked by *p*-value. (**B**) The top canonical pathways ranked by absolute activation z-scores (right panel) and the numbers of up- or down-regulated genes in each pathway (left panel). Z-scores predict the activation or inhibition of each individual pathway.

To uncover the molecules and biological pathways associated with cellular responses to ssUVR, Gene Ontology (GO) for the significant DEGs was analyzed using Database for Annotation, Visualization and Integrated Discovery (DAVID), an NCBI web-based functional annotation tool. The top 10 highly represented biological processes of down-regulated genes, shown in Fig. 2A, are mainly related to mitotic cell cycle regulation and glycolysis, with each group containing 4/10 and 2/10 GO terms respectively. The four GO terms that are related to mitotic cell cycle regulation are mitotic nuclear division (p = 1.71 × 10^–17^), mitotic cytokinesis (p = 2.93 × 10^−10^), sister chromatid cohesion segregation (p = 4.02 × 10^−8^) and spindle organization (p = 2.63 × 10^−6^); while the two GO terms that are related to glycolysis are glycolytic process (p = 2.72 × 10^−8^) and canonical glycolysis (p = 1.86 × 10^−6^). In contrast, GO terms from genes that were up-regulated by ssUVR are quite diverse. The top three GO terms for the upregulated genes are rRNA processing (p = 6.32 × 10^−6^), stress response (p = 3.31 × 10^−5^) and extracellular matrix organization (p = 3.31 × 10^−5^) (Fig. 2A).

Ingenuity Pathway Analysis (IPA) was used to identify major pathways that were enriched in the DEG sets. Consistent with DAVID analysis, pathways related to cell cycle regulation were overrepresented (Fig. 2B). The top two canonical pathways are G2/M phase DNA damage checkpoint regulation (AURKA, BORA, CCNB1, CCNB2, CDC25C, CDK1, CKS2, CKS1B, PLK1, TOP2A) and mitotic roles of Polo-like kinase (CCNB1, CCNB2, CDC20, CDC25C, KIF23, PLK1, PRC1, PTTG1, RAD1). In addition, the genes involved in G1/S checkpoint regulation, regulation of cellular mechanics by calpain protease, and ATM signaling were also overrepresented. Interestingly, the majority of genes related to cell cycle regulation are down-regulated, especially those involved in mitotic-related spindle assembly checkpoint, such as BUB1, BUB1B, CCNB1, CCNB2, CDC20, CDK1, PLK1, PTTG1. Other highly enriched pathways are those related to cell adhesion and important cell signaling pathways, such as Neuregulin signaling, NF-kB signaling, IL-1 signaling, death receptor signaling and p38 MAPK signaling.

In addition to canonical pathway analysis, upstream regulator analysis (IPA) was used to predict the activation or inhibition of upstream regulators based on the Ingenuity Knowledge Base. Table 1 lists the transcription factors that were predicted to be either activated or inhibited in upstream regulator analysis. HaCaT cells exposed to single ssUVR exhibited significant inhibition of FOXM1, ATF6, FOXO1, MITF, ATF4, and EHF. FOXM1 is a transcription factor regulating the expression of genes essential for DNA damage repair, cell cycle progression, and mitosis^27,28^. ATF4 and ATF6 are transcription factors that regulate expression of genes involved in endoplasmic reticulum (ER) stress response^29^. While ATF6 acts as a sensor for ER stress and controls the expression of genes required for the unfolded protein response, ATF4 plays important role in restoring ER homeostasis and regulation of genes involved in autophagy^29–31^. Moreover, KDM5B, TP63, SMAD4, CDKN2A, NUPR1, Notch1 and STAT3 are predicted to be highly activated regulators (Table 1).

**Table 1:** Top activated and inhibited upstream regulator in cells exposed to single ssUVR.

Upstream Regulator	Predicted Activation State	Activation z-score	Target molecules in dataset
FOXM1	Inhibited	−3.208	AURKB, BUB1B, CCNA2, CCNB1, CCNB2, CDC20, CDC25C, CDCA2, CDCA8, CDK1, CDKN1B, CDKN3, CENPA, CKS1B, CKS2, KIF20A, LDHA, MMP2, MYC, NEK2, PGK1, PLK1, PRC1
ATF6	Inhibited	−2.433	AURKA, BUB1, CDKN3, HMMR, PVR, UBE2C
FOXO1	Inhibited	−2.353	ANLN, CCNB1, CCNB2, CDK1, CDKN1B, DEPDC1, DLGAP5, IER3, JAG1, MYC, NEK2, NUSAP1, PRC1, TXNIP
MITF	Inhibited	−3.638	AURKB, CCNB1, CCNF, CD44, CDCA8, CEP55, KIF20A, KIF4A, KIFC1, NCAPD2, NUF2, PLK1, SERPINE1, SPAG5, TPX2, TXNIP, UBE2C
ATF4	Inhibited	−2.193	ASNS, CEBPB, DDIT4, PCK2, PSAT1
EHF	Inhibited	−2.309	CDK1, CDKN1B, CRABP2, EHF, JAG1, KLK8, S100A9, SAA1, SCEL, SERPINE1, SPRR1B, ZFP36L1
STAT3	Activated	2.197	DKK1, JAG1, MMP2, MYC, SNAI2
NOTCH1	Activated	2.012	CD44, CDKN1B, EFNB2, EGFR, MYC, NRG1, PTPRK
NUPR1	Activated	2.714	ABCC5, ALDOC, AREG, AURKA, BRI3BP, BTG1, BUB1, BUB1B, CCNA2, CCNB2, CCNF, CDC25C, CDCA2, CDCA8, CEBPB, CKAP2L, CYR61, FAM162A, GPSM2, HJURP, ITPR3, KIF20A, KIF23, KIF2C, KIFC1, MAT2A, MCM10, MXD1, MYC, NDRG1, NR1D2, NUCKS1, PHLDA1, PLK1, PRNP, SERPINE1, SLC2A1, SPAG5, SRSF1, TMEM19, TNIP1, TRAFD1, TRIB1, ZC3HAV1, ZFP36L1
CDKN2A	Activated	2	CCNA2, CCNB1, CDC25C, SERPINE1
SMAD4	Activated	2.424	FSTL3, IER3, JAG1, SERPINE1, SNAI2, THBS1
TP63	Activated	2.933	CCNA2, CDC25C, CDK1, CDK6, CDKN1B, CKS2, CYR61, DKK1, DUSP6, EGFR, F3, FDXR, FGFR3, FST, FUBP1, GPX2, HBP1, HIRA, ID3, IER3, IGFBP3, IL1B, ITGA2, JAG1, KIF23, KRT6A, MCM10, MPZL2, MYC, NT5E, PRNP, PTHLH, PTPN12, RACGAP1, THBS1, TIPIN, TNS4
KDM5B	Activated	3.12	ARL6IP5, AURKA, BUB1B, CCNB1, CDK1, DLGAP5, EPB41L1, HMMR, ISG15, KIF2C, MCM3, NDC80, NMB, PIR, TOP2A, TTK

Taken together, the analyses of enriched pathways and upstream regulators revealed a significant inhibition of cell cycle progression and the expression of stress-related genes are among the most important events occurred at 24 hours after ssUVR.

### Functional analysis of DEGs in HaCaT cells exposed to 5 repetitive ssUVR

**Figure 3:**
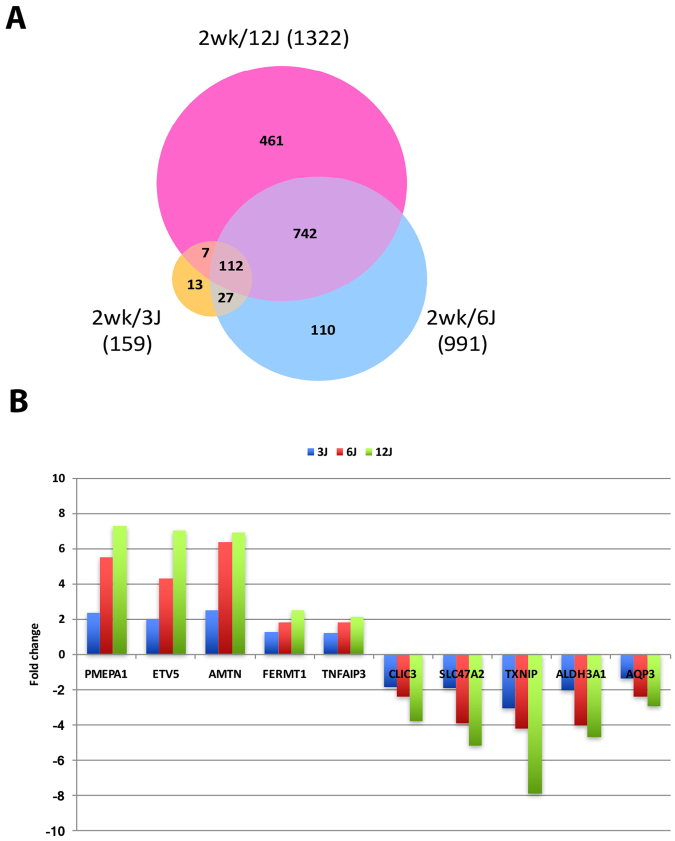
Differentially expressed genes in cells exposed to 5 repetitive ssUVR at 3, 6, 12 J/cm^2^. (**A**) Venn diagram of differentially expressed genes in cells exposed to 3, 6, and 12 J/cm ssUVR. (**B**) Illustration showing dose-dependent changes of 5-upregulated and 5-downregulated genes in cells exposed to 5 repetitive ssUVR.

In HaCaT cells exposed to 5 repetitive doses of ssUVR, the number of DEGs was correlated to the increased intensity of ssUVR. While cells exposed to 3 J/cm^2^ had 159 genes significantly changed, the numbers increased to 991 in cells exposed to 6 J/cm^2^ and 1322 in those with 12 J/cm^2^ (Supplemental Table 1). Among all of the genes changed after 5 repetitive exposures to ssUVR, 60% of the DEGs identified overlapped in two or three dose groups. The ratio of overlapping genes to total changed genes were 92% in the group exposed to 3 J/cm2, 89% in those exposed to 6 J/cm2, and 65% in cells exposed to 12 J/cm^2^ (Fig. 3A). Moreover, 92 out of 112 genes that overlapped in all three dose groups exhibited a dose-dependent fold change (FC). Among these genes, dose-dependent changes of 10 representative genes (5 up- and 5 down-regulated genes) are illustrated in Fig. 3B. Thus, the results suggest that not only the number of DEGs but also the fold change of each DEG was correlated to increased intensity of ssUVR.

**Figure 4:**
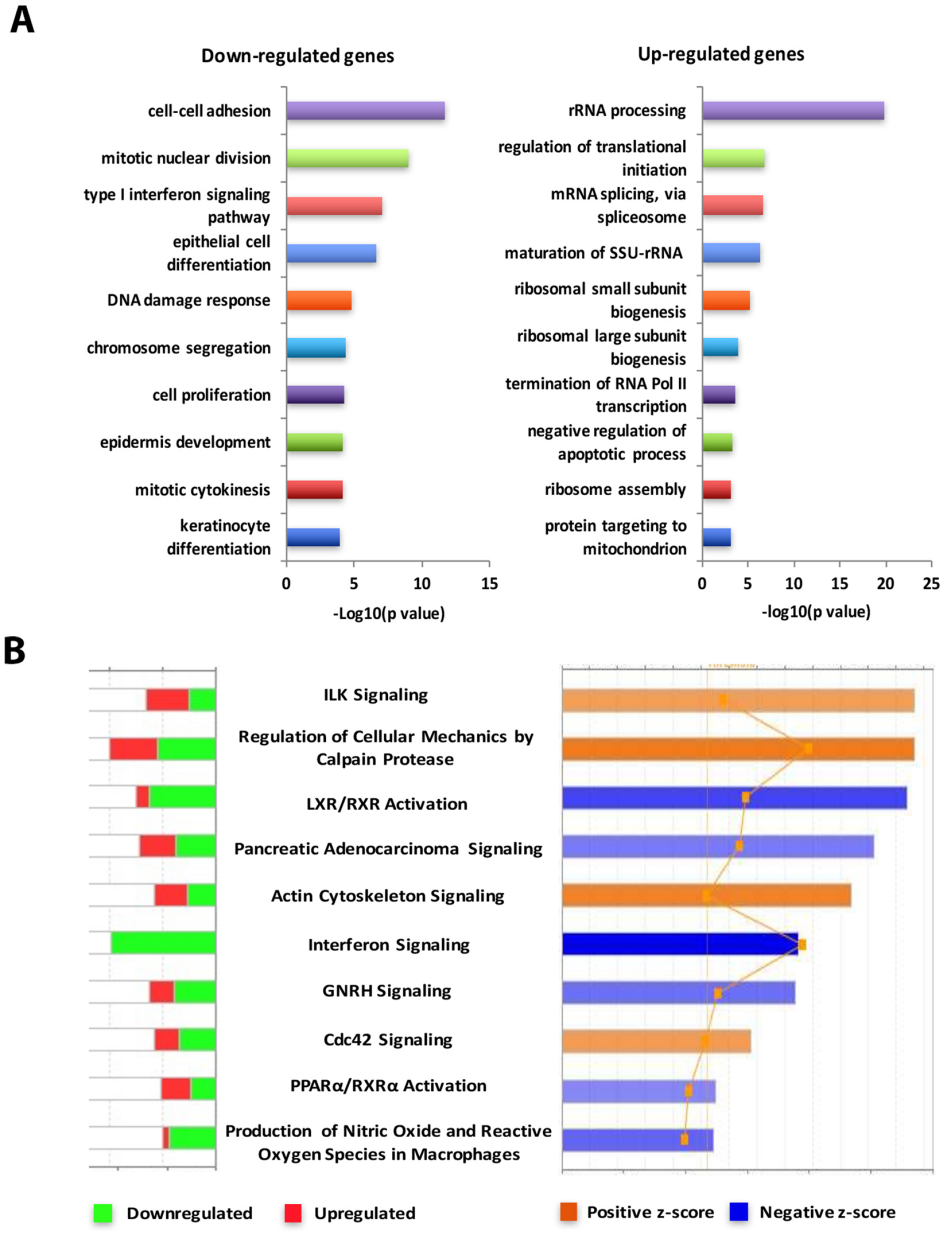
Functional annotation and pathway analysis of DEGs in HaCaT cells exposed to 5 repetitive ssUV radiation at 12 J/cm^2^. (**A**) The top biological process GO terms of down- (left panel) and up- (right panel) regulated DEGs ranked by *p*-value. (**B**) The top canonical pathways ranked by absolute activation z scores (right panel) and the numbers of up- or down-regulated genes in each pathway (left panel). Z scores predict the activation or inhibition of each individual pathway.

To study the functional relevance, the DEGs identified in the highest dose of ssUVR (12 J/cm^2^) were subjected to DAVID annotation and IPA analysis. As shown in Fig. 4A, while cell cycle regulation remains the major GO term category in down-regulated genes, including the terms mitotic nuclear division (p = 1.06 × 10^−9^), chromosome segregation (p = 4.16 × 10^−5^), and mitotic cytokinesis (p = 7.68 × 10^−5^); the rest of top 10 GO terms represent a diverse biological function, including cell-cell adhesion (p = 2.18 × 10^−17^), type I interferon signaling (p = 8.01 × 10^−8^), DNA damage response (p = 1.69 × 10^−5^), epithelial cell differentiation (p = 2.63 × 10^−7^), and epidermis development (p = 7.53 × 10^−5^). Surprisingly, the majority of highly enriched GO terms in the up-regulated genes are related to protein translation, including rRNA processing (p = 1.66 × 10^−20^) and ribosomal biogenesis (p = 6.83 × 10^−6^). Canonical pathway analysis revealed that integrin-linked kinase (ILK) signaling, an important pathway mediating cell-cell and cell-matrix interaction^32^, was the top overrepresented canonical pathway for this exposure group(Fig. 4B). Several members of integrin receptors (ITGA2, ITGAV, ITGB1, ITGB5 and ITGB6) were increased in ssUV irradiated cells. Moreover, the calculated z-score predicted a significant inhibition of genes involved in interferon signaling as well as nuclear receptor-mediated pathways (LXR/RXR and PPAR/RXR). Interestingly, a significant downregulation of both type I and type II IFN pathways in cells exposed to repetitive ssUVR was observed. Both STAT1 and STAT2 were reduced in cells exposed to ssUVR, accompanied by the downregulation of IFN target genes, including IRF1 and IFITM1-3 (Supplemental Fig. 2). Lastly, upstream regulator analysis revealed a significant inhibition of TP73 and Nrf2 activity along with the activation of c-Myc, ATF3 and CBX5, which represented altered upstream transcriptional regulation in the cells exposed to 5 repetitive ssUVR (Table 2).

**Table 2:** Top activated and inhibited upstream regulator in cells exposed to 5 repetitive ssUVR.

Upstream Regulator	Predicted Activation State	Activation z-score	Target molecules in dataset
FOXM1	Inhibited	−2.933	AURKB, BUB1B, CCNA2, CCNB2, CDCA2, CDCA8, CDK1, CDKN1A, CDKN1B, CENPA, CENPF, CKS1B, CKS2, KIF20A, MMP2, NEK2, PGK1, PRC1
TP73	Inhibited	−2.744	AUH, CAMLG, CDC42EP2, CDH1, CDK1, CDKN1A, CDKN1B, CXCL1, FASN, FGFR3, HES1, HSPA1A/HSPA1B, IGFBP3, IGSF3, JAG2, KLHL21, PMAIP1, PPL, RB1CC1, SAT1, SERPINE1, SFN, SHISA5, SNAI2, THBS1, TNFRSF10A, TNFRSF10B, TNFRSF1A, TSPAN1
NFE2L2	Inhibited	−2.959	CAT, CDH1, FTH1, FTL, GCLC, GCLM, IL1RN, KEAP1, SERPINE1, SHC1, SNAI2
MITF	Inhibited	−3.273	AURKB, CCNF, CD44, CDCA3, CDCA8, CENPF, ECT2, EME1, ESPL1, KIF20A, KIF4A, KIFC1, MGAT4B, NCAPD2, NUF2, SERPINE1, SPAG5, TPX2, TXNIP, UBE2C, UPP1
EHF	Inhibited	−2.828	ALOX5, CDK1, CDKN1B, CRABP2, E2F1, EHF, IL1RN, JAG1, KLK8, MUC1, PPARD, S100A9, SAA1, SAA2, SCEL, SERPINE1, SPRR1B, THBD
STAT3	Activated	2.124	CD46, CDH1, CDKN1A, DKK1, EME1, FN1, HAS2, IL1RN, IRF1, ITGAV, ITGB1, JAG1, MMP2, MUC1, NOTCH1, PMAIP1, SNAI2, STAT1
NUPR1	Activated	3.108	ABCC5, AGRN, ALDOC, AURKA, BUB1, BUB1B, CCNA2, CCNB2, CCNF, CDCA2, CDCA3, CDCA8, CKAP2L, CYR61, DHTKD1, DUSP5, EME1, ERMP1, ESPL1, ETS1, FAM162A, FAM72C/FAM72D, FOXO3, HBEGF, HJURP, HNRNPM, IP6K2, ITPR3, KIF11, KIF20A, KIF2C, KIFC1, KNL1, LMNB1, LOC102724428/SIK1, MAT2A, MCM10, MKI67, MSANTD3, MXD1, MYO18A, NAA40, NDRG1, NGFR, NUCKS1, OSER1, PAGR1, PEA15, PFKFB3, PHLDA1, POLA2, PRNP, RAB38, SAT1, SERPINE1, SERTAD2, SIGMAR1, SLC2A1, SPAG5, SRSF1, TOB1, TRAFD1, TRERF1, TRIB1, TRIM16, UAP1, UNC5B, UPP1, XBP1, ZFAND2A, ZNF488
CBX5	Activated	2.828	AHSA2, AKR1C3, ALDH3A1, CD24, CYP1B1, FGFBP1, FOXQ1, MAL2, MMP7, PROM2, PTK6, RARRES3, RIN2, TACSTD2, TGFBI, TM4SF1, TSPAN13, TXNIP
ATF3	Activated	2.156	AURKA, AURKB, CD82, CDK1, ID1, LDLR, NEK2, SERPINE1, TNFRSF10B
SMAD4	Activated	2.792	CDKN1A, ERCC5, FSTL3, IER3, JAG1, JAG2, KLK11, MUC4, SERPINE1, SHISA5, SNAI2, THBS1, TIMP3, TNFRSF10A
MYC	Activated	2.748	APEX1, ATP13A2, CAD, CD44, CDK4, CDKN1A, CDKN1B, CLU, CTDSPL, DCTPP1, DDB2, DKC1, E2F1, FASN, GCLC, GCLM, GJA1, GLS, GLUD1, GLYR1, GPI, GSR, HNRNPU, HSPB1, HSPD1, HSPH1, IDH1, JUN, NCL, NOLC1, NOP56, NPM1, ODC1, PDCD4, PERP, PIAS3, PMAIP1, POLDIP3, RANBP2, SAT1, SCPEP1, SHMT1, SLC2A1, SRSF1, TMEM126A, TMSB10/TMSB4X, TNFRSF10A, TNFRSF10B, TXNIP, UBE2C

### Comparisons of genes and pathways in response to different ssUVR

**Figure 5:**
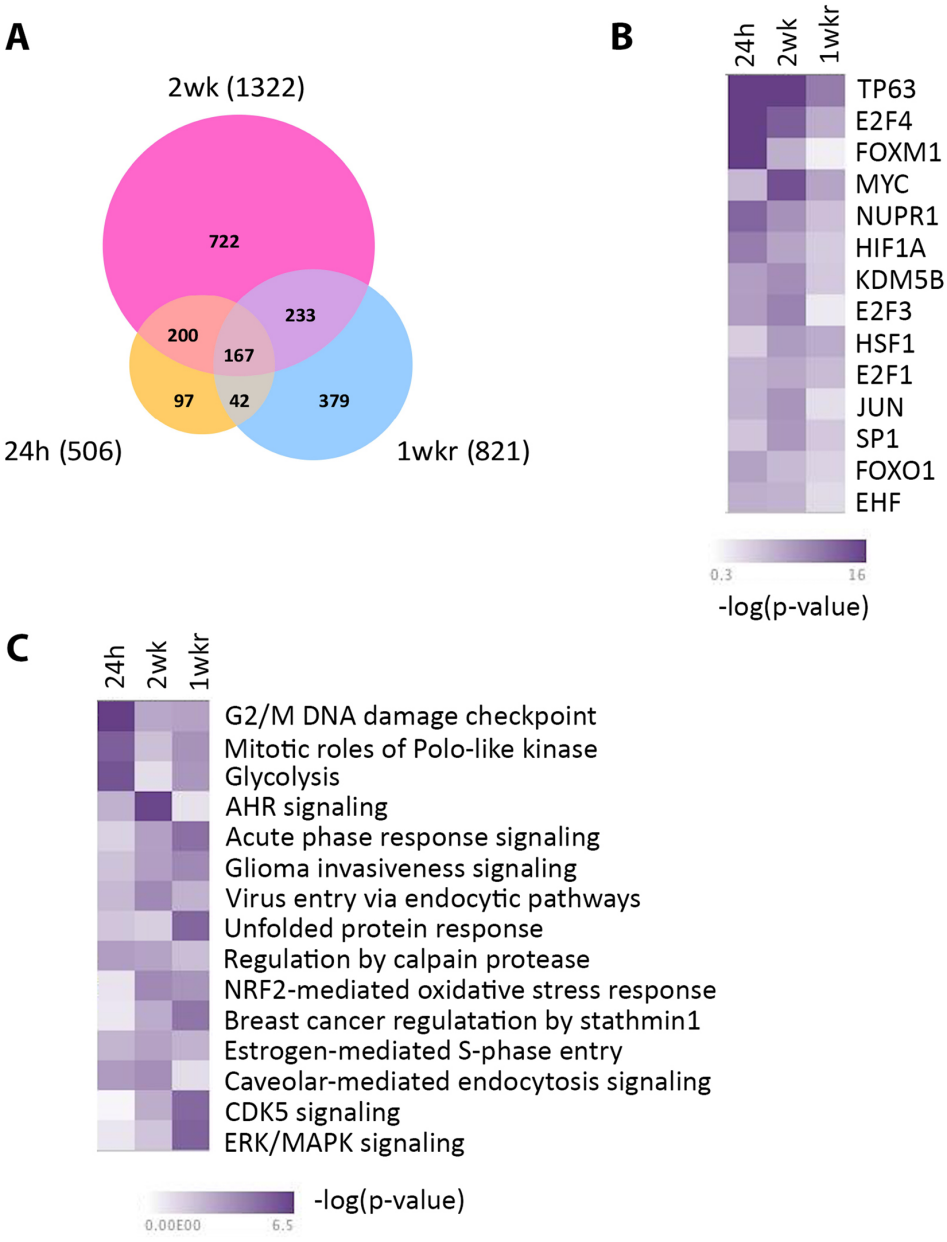
Comparison of genes and pathways changed in HaCaT cells exposed to signle ssUVR, 5 repetitive ssUVR, or 1-week recovery from repetitive ssUVR. Venn diagram of differentially expressed genes (**A**), predicted upstream transcription factors (**B**), and the top canonical pathways (**C**) were shown.

To examine the persistence of the changes in gene expression by ssUVR exposure, gene expression changes were analyzed in cells that were collected at one week after the 5^th^ ssUVR exposure (12 J/cm^2^). A total of 821 genes were altered with treatment, including 505 up-regulated genes and 316 down-regulated genes (Supplemental Table 1). Interestingly, about 400 DEGs from the 1 week recovery group were overlapped with those from cells collected 24 hours post irradiation, suggesting that nearly half of ssUVR-induced gene alterations persist for several cell generations. Cross-comparison of the 3 DEG lists yielded a total of 167 genes that were commonly altered in all three sample sets (Fig. 5A). After removing 8 genes that were regulated in an opposite direction, we obtained a list of 159 DEGs (84 up-regulated and 75 down-regulated) that were altered by both single and repetitive ssUVR and retained the persistent change even at 1 week after irradiation (Supplemental Table 2).

A comparative analysis of the three DEG list was performed using the IPA comparison analysis tool in order to further investigate the alteration of genes or pathways in cells exposed to single or 5 repetitive ssUVR as well as cells undergoing a 1-week recovery. As shown in Fig. 5B, the comparison analysis of upstream regulators revealed two distinct groups of transcription factors. The first group is overrepresented in cells exposed to single ssUVR, and included TP63, E2F4, FOXM1, NUPR1 and HIF1A. Among these TFs, TP63 and E2F remained highly represented in cells with 5x ssUVR, while others were less prominent. In contrast, the second group of TFs were more likely changed only after repetitive ssUVR. The transcription factors in this group contain MYC, KDM5B, E2F3, JUN and SP1. Interestingly, these TFs seemed less represented in cells following 1 week recovery, suggesting a relatively transient TF activity after ssUVR. The shift in expression of transcriptional regulators from group I to group II may reveal the transition of cellular response from early cell cycle arrest and stress response toward to a more sophisticated adaptation. Comparative analysis of canonical pathways also revealed the transition of cellular response from cell cycle regulation toward metabolic changes mediated by AHR/NRF2 (Fig. 5C). It is worth noting that, unfolded protein response, CDK5 signaling and ERK/MAPK signaling were overrepresented in the 1 week recovery group.

## Discussion

Despite many studies on UVA/B/C radiation-induced changes in gene expression, the molecules and pathways involved in the cellular response to low dose sunlight are incompletely understood. In the present study, we investigated the genes and pathways altered in response to single dose or repetitive solar simulated UV irradiation of HaCaT keratinocytes using RNA-seq and subsequent functional annotation tools. These results reveal that ssUVR is able to up- or down-regulate genes with diverse cellular functions in a dose-dependent manner. While cells exposed to single dose of ssUVR displayed a significant inhibition in genes related to cell cycle progression, especially mitotic related genes, cells that were exposed to repetitive doses of ssUVR exhibited changes in genes involved in signal transduction, cell structure and metabolism. In addition, we identified a list of ssUVR target genes, which were altered early in the response to ssUVR and maintained the changes for several cell generations post irradiation.

Mammalian cells have developed a sophisticated response to protect themselves against DNA damage induced by UV radiation. Multiple checkpoints are activated during the cell cycle to pause cell division at a specific phase until the damage repair is completed or bypassed^33^. While G1/S and G2/M checkpoints control the entry into S-phase and M-phase respectively, intra-S and mitotic spindle checkpoints maintain the fidelity of DNA replication and chromosome segregation. These checkpoints are controlled by distinct yet interconnected pathways and gene networks. Our study revealed that DEGs related to cell cycle regulation were overrepresented in keratinocytes exposed to a single dose of ssUVR. Although the changes covered all four checkpoints and cell cycle phases, the majority of down-regulated genes were related to G2/M arrest and mitotic spindle checkpoint regulation.

Transition from G2 phase into mitosis is driven by CDK1 (Cdc2) and its regulator cyclin B. During G2 phase, CDK1 is inactivated by phosphorylation of T14 and Y15 by protein kinases WEE1 and MYT1^34,35^. Activation of CDK-Cyclin B involves dephosphorylation of CDK1 by CDC25 phosphatase^36^ and phosphorylation of Cyclin B by PLK1^37^. PLK1 is a serine/threonine protein kinase that plays important roles in mitosis as well as DNA damage response^38,39^. During G2/M transition, PLK1 is activated by Aurora A kinase and its cofactor Bora^40^. Upon detection of DNA damage, activation of the ATM/ATR/CHK1/CHK2 signaling cascade leads to inhibition of CDC25C and BORA/ Aurora A/PLK1, which subsequently inactivates CDK1-Cyclin B leading to G2/M cell cycle arrest^38,39^. Although most of the regulatory events occur through phosphorylation and dephosporylation, it was previously reported that CDC25C protein levels were reduced in HaCaT cells exposed to UVB^41^. Our results showed a significant down-regulation of these genes in ssUV-irradiated HaCaT cells. It is worth noting that HaCaT carries p53 mutations in both alleles and therefore does not have a functional p53 protein. In cells that do not have functional p53, cell arrest at the G2/M boundary has been reported to be mediated by reduced CDC25C and subsequent inactivation of the cyclin B1-CDK1 complex^41^. The down-regulation of CDC25C, cyclin B1, cyclin B2 and CDK1 identified in our study may be specific to mitotic arrest in HaCaT cells exposed to ssUVR. Despite the lack of functional p53, the changes of many genes involved in the mitotic process identified in our HaCaT cells exhibited similar regulation when compared to UV irradiated skin cell *in vivo* ^11^.

The second most significant canonical pathway predicted by IPA in cells exposed to single ssUVR is the mitotic roles of the polo-like kinase. In addition to its role in G2/M checkpoint and mitotic entry, PLK1 activity is crucial for nearly every step of mitosis, including spindle assembly, mitotic exit and cytokinesis^38,39^. Consistent with reduced PLK1 protein levels, a large number of mitosis-related genes were also down-regulated in cells exposed to single ssUVR, such as mitotic related kinases (AURKA, AURKB, BUB1, BUB1B, PLK1, TTK, NEK2, INCENP) and genes involved in spindle assembly, chromosome segregation, as well as cytokinesis (CDC20, HAUS4, KIF22, KNTC1, ANLN, PTTG1, FAM83D, KIF2C, NUMA1, KLHL21, KIF11, CKAP5, TPX2, NUF2, CENPF, NDC80, PBK, REEP4, ARL8A, MIS18BP1, SNX33, etc.). The massive down-regulation of mitotic related genes in ssUV irradiated cells is likely due to the activation of the G2/M DNA damage checkpoint. This is in line with previous findings of delayed expression of mitotic regulators in cells exposed to ionizing radiation^42^. Interestingly, a recent study reported that the expression of mitotic genes is up-regulated in fibroblasts exposed to low dose UVC^19^. It is not clear whether this upregulation is specific to UVC or fibroblasts, as UVR-induced downregulation of mitotic genes has been reported in both cultured keratinocytes as well as human epidermis^11,43^. A recent study investigated the different impact of longwave UVA (UVA1) exposure in keratinocytes and dermal fibroblasts using the reconstructed skin model^44^. Transcriptome analysis of keratinocytes and dermal fibroblasts of reconstituted skin revealed that less than 22% of UVA1-modulated genes were commonly altered in both cell types, suggesting a cell type-specific transcriptome changes in response to UVA1 exposure^44^. The cell type-specific response is not only limited to gene expression but also the UV radiation-induced stress response. While a low dose of UVA1 induced apoptosis in dermal fibroblasts, epidermal keratinocytes showed no apoptotic morphology, despite the clear evidence of DNA damage and ROS production in both cell types^44^. In support of their findings, our study showed that the expression of genes related to cell death and apoptosis were not significantly altered in ssUVR exposed HaCaT cells.

In HaCaT cells exposed to repetitive ssUVR, the most inhibited canonical pathway, predicted by IPA analysis, was interferon signaling. Interferon signaling pathways are crucial for antiviral and antibacterial defense^45^. There are three types of interferons; Type I interferons contain IFN-α, IFN-β, IFN-ε, IFN-κ, and IFN-ω; Type II included IFN-γ and type III contains IFN-λ^45^. Type I IFNs bind to IFN-α receptor I and II, which subsequently activate STAT1 and STAT2 by phosphorylation and activation of janus kinase 1 (JAK1) and tyrosine kinase 2 (TK2)^45,46^. Activated STAT1 and STAT2 complexes translocated to the nucleus and bound to IFN-stimulated response elements, activating type I IFN-response genes. Type II IFNγ bound to cell surface receptor IFNγR and activated STAT1 homodimers and respective downstream target genes. Many studies have shown that type I interferons are key players in normal skin and are normally downregulated in three major types of skin cancer: squamous carcinoma, basal cell carcinoma, and melanoma^46–48^. Our results show that there was a significant downregulation of type I and type II IFN pathways in cells exposed to repetitive ssUVR. Both STAT1 and STAT2 were reduced in UV radiated cells, accompanied by downregulation of IFN target genes, including IRF1 and IFITM1-3. IRF1 and IRF6 were also down-regulated in the irradiated cells. This result is consistent with a previous study that UVR is able to down-regulate IFNγ activated STAT1 phosphorylation in both human and mouse keratinocytes^49,50^. IRF-6 is highly expressed in skin, and is crucial for skin and limb formation as well as craniofacial development. Depletion of IRF6 strongly affected keratinocyte proliferation and differentiation^51^. UVR-induced downregulation of IFN pathways and their respective target genes were not observed in cells exposed to single ssUVR, suggesting that the changes may be a late response that requires repetitive UV exposure. In addition to interferon signaling, multiple nuclear receptors were also down-regulated by ssUVR, such as RARr, RXRa and RXRb. Both LXR/RAR and PPAR/RXR activation were predicted to be inhibited following ssUVR exposure. Moreover, genes involved in cell migration and cytoskeleton reorganization were induced, such ILK signaling and CDC42 signaling. Therefore, compared to a strong inhibition of cell cycle progression in cells exposed to single ssUVR, cells exposed to repetitive ssUVR displayed a variety of changes in cell signaling and metabolism.

UV radiation, particularly at shorter wavelength, is able to modulate the skin neuroendocrine activity^6^. Several studies have reported an increase of cortisol synthesis by UVB/UVC, which was associated with the increased levels of CRH, POMC, 11βHSD1, CYP11A1, CYP11B1, MC1R, MC2R and decreased 11βHSD2^52–55^. Some genes were altered in a similar trend in our study, but the changes were not statistically significant (data not shown). It is worth noting that the UV source used in our study consists of 95% UVA and 5% UVB. This small amount of UVB may not be sufficient to induce significant gene changes related to cortisol synthesis, as UVA alone had no effect on local cortisol production^6,53^.

Our previous study demonstrated a massive reduction of histone lysine acetylation in human keratinocytes exposed to ssUVR^24^. As histone acetylation is normally associated with active gene expression, it is quite surprising that the numbers of up-regulated and down-related genes are equally represented in most comparison conducted in our study, with the exception of cells exposed to repetitive ssUVR at 3 J/cm^2^. The data suggests that histone hypoacetylation may have a very limited impact on ssUVR-altered gene expression. This is in line with a recent study using HEK293 cells which demonstrated limited correlation between genomic distribution of H4K16 acetylation and transcriptional regulation^56^. It is possible that ssUVR-induced histone hypoacetylation leads to changes in chromatin structure and contributes to both DNA damage sensing and successful DNA repair, rather than the global changes of gene expression. However, we do not exclude the possibility that altered histone acetylation may mediate ssUVR-induced expression changes in a gene-specific manner. For example, aquaporin 3 (AQP3), an important cell membrane protein that is crucial for water and glycerol transportation as well as skin hydration^57,58^, was downregulated in cells exposed to either single ssUVR (−1.9 fold) or repetitive ssUVR (−2.9 fold) (Supplemental Table 2). These results were consistent with the finding of AQP3 downregulation and decrease of water permeability in keratinocytes exposed to UVB^59^, Interestingly, AQP3 levels remained low in cells undergo 1-week recovery (Supplemental Table 2), suggesting a possible epigenetic change mediates AQP3 down-regulation by ssUVR. This was further supported by a recent study on HDAC3 suppression of AQP3 expression in mouse epidermal keratinocytes^60^.

In summary, our present study compared changes in gene expression in human keratinocytes exposed to single or repetitive ssUVR. In addition, changes present one week after the final dose of repetitive irradiation were measured. Using RNA-seq combining with IPA comparative analysis, DEG lists were generated and pathways and their connected upstream regulators were analyzed to look for changes in expression resulting in cellular abnormalities. The results indicate that ssUVR-modulated the expression of genes with diverse cellular functions. While single ssUVR caused a significant inhibition of genes involved in cell cycle progression, repetitive ssUVR lead to extensive changes of genes related to many important cell signaling pathways, cell adhesion, and metabolism. These data suggest that a complex network of transcriptional regulators and pathways orchestrate the cellular response to ssUVR. Moreover, we identified 159 genes that maintained their initial changes after several cell passages, suggesting a potential epigenetic mechanism that may mediate ssUVR-induced transcriptome changes. Further analysis of the epigenetic landscape in cells exposed to single or repetitive ssUVR will provide new insights into the mechanism underlying solar UV radiation-induced skin damage and carcinogenesis.

## Methods

### Cell culture

Immortalized human HaCaT keratinocytes were obtained from Dr. Chuangshu Huang at NYU School of Medicine. The cells were cultured in DMEM medium (4.5 g/L glucose) containing 10% fetal bovine serum and 1% penicillin/streptomycin at 37 °C and 5% CO_2_.

### Solar simulated UV radiation

The UV source and simulated UV radiation are the same as previously described^24^. Solar simulated UV radiation was performed using a modified Hand Foot II phototherapy instrument (National Biological Corporation, Beachwood, OH) with 8 UVA lamps (HOUVALITE F24T12/BL/HO [PUVA], National Biological Corporation). The lamp emission is filtered by a single glass plate, resulting in an average intensity of 3.4–3.5 mw/cm^2^ for UVA and 0.18 mw/cm^2^ for UVB at cell culture surface, which equivalent to a spectrum of 95% UVA and 5% UVB. UVA intensity was measured with a UVX digital handheld radiometer using the UVX-36 UVA sensor (UVP, Upland, CA). UVB intensity was measured with an ILT1400 Photo detector using the SEL240 UVB sensor (International Light, Newburyport, MA). The intensity of the lamp output was measured before every exposure and the exposure times were calculated to deliver the desired doses. The lowest dose (3 J/cm^2^) and the highest dose (12 J/cm^2^) used in this study required the approximate exposure time of 15 min and 57 min, respectively.

For cells exposed to single ssUVR, 3.5 × 10^6^ cells were seeded in 10-cm cell culture dish at the day before irradiation. Prior to exposure, cells were washed twice with PBS and exposed to 12 J/cm^2^ ssUVR in 10 ml of Hank’s Balanced Salt Solution (HBSS, life technologies, Grand Island, NY) supplemented with 4 mM glucose. After irradiation, the HBSS was replaced with fresh medium and cells were cultured for 24 hours. Control samples were treated identically but covered with foil during irradiation (Sham). To maintain a constant temperature, a table fan was used to reduce heat production during the exposure. For repetitive radiation, cells were exposed to 5 repetitive doses of ssUVR at 3, 6, 12 J/cm^2^ as described above, except they were allowed to recover for 3 days prior to the next round of radiation. To examine the persistence of gene expression changes, cells were exposed to 5 repetitive doses of ssUVR at 12 J/cm^2^, and were harvested at one week after final irradiation.

### RNA isolation and sequencing library preparation

Total RNA was extracted from sham and ssUVR-exposed cells using the Trizol reagent (Life Technologies, Gaithersburg, MD). RNA-Seq libraries were prepared using Illumina TruSeq RNA-sample preparation kit according to the manufacture’s instruction. Sequencing was performed at the NYU School of Medicine Genome Technology Center using Illumina HiSeq. 2500 by multiplexed single-read run with 50 cycles.

### RNA-Seq data analysis

Raw sequence data (Fastq) were loaded into Biomedical Genomics Workbench Version 3.5.3 (Qiagen) for data analysis. The raw Fastq files were trimmed to remove any remaining adaptors and ambiguous nucleotides. The trimmed sequence files were aligned to human genome (Hg38) allowing two mismatches. Reads mapped to the exons of a gene were summed at the gene level. Gene expression levels were quantified as total read per million (TPM). Differential gene expression was analyzed using Advance RNA-seq plug-in tool (Qiagen) by comparing each treated group versus corresponding control group. TMM (trimmed mean of M values) normalization was performed to adjust library sizes before differential expression analysis. The genes with a false discovery rate (FDR) < 0.05 between control and treated group and an average mean of total counts no less than 10 were defined as differentially expressed genes (DEGs).

### Gene ontology and pathway analysis

Functional annotation was analyzed with the Gene Ontology (GO) classification system using the Database for Annotation, Visualization and Integrated Discovery (DAVID), an NCBI web based functional annotation tool. Gene network, top pathways and upstream regulators were analyzed by Ingenuity Pathway Analysis (Qiagen).

### RT-PCR validation

Total RNA was isolated with TRIzol Reagent (Life Technologies, Gaithersburg, MD). Reverse transcription was performed using SuperScript™ III First-Strand Synthesis System for RT-PCR (Invitrogen, Carlsbad, CA). Real time-PCR was performed by using SYBR Select Master Mix (Applied Biosystems, Foster City, CA) on a 7900HT Fast Real-time PCR System (Applied Biosystems). Each sample was run in triplicates. The relative mRNA expression levels were normalized by using *GAPDH* as the endogenous control. The primers used for qPCR were as follows: TXNIP forward: 5′-ACTCGTGTCAAAGCCGTTAGG-3′; TXNIP reverse: 5′-TCCCTGCATCCAAAGCACTT-3′; DDIT4 forward: 5′-CGGGTGGCTGTGCATTG-3′; DDIT4 reverse: 5′-TGGCCCCTAAGCCTTTGTT-3′; PMEPA1 forward: 5′-TCTCTGCAGGGCGCTTTG-3′; PMEPA1 reverse: 5′-GTTACCGCACCTGCCTTCAC-3′; KRT4 forward: 5′-AACACCCGGCAGCACTTG-3′; KRT4 reverse: 5′-GGAGTAGAATGGGACACTAAGTGGTT-3′.

### Data Availability Statement

The datasets generated during the current study have been deposited in NCBI Gene Expression Omnibus (GEO accession number GSE102676).

## Electronic supplementary material

Supplemental Figures

Supplemental Table 1

Supplemental Table 2
