# Supplementary material for: Transcription factors and stress response gene alterations in human keratinocytes following Solar Simulated Ultra Violet Radiation
Source: Sci Rep. 2017 Oct 19;7:13622. doi: 10.1038/s41598-017-13765-7 (PMC5648893; doi:10.1038/s41598-017-13765-7)
Supplement: Supplementary file 1 — Supplemental Figures [file 41598_2017_13765_MOESM1_ESM.pdf]

Transcription factors and stress response gene alterations in human keratinocytes following Solar Simulated Ultra Violet Radiation

Thomas L. Des Marais[1], Thomas Kluz[1], Dazhong Xu[3], Xiaoru Zhang[1], Lisa Gesumaria[1], Mary S. Matsui[2], Max Costa[1]*, Hong Sun[1]*

[1]New York University, Department of Environmental Medicine, Tuxedo, New York, United States of America.

[2]Estee Lauder Companies, Inc., Melville, New York, United States of America.

[3]New York Medical College School of Medicine, Department of Pathology, Valhalla, New York, United States of America.

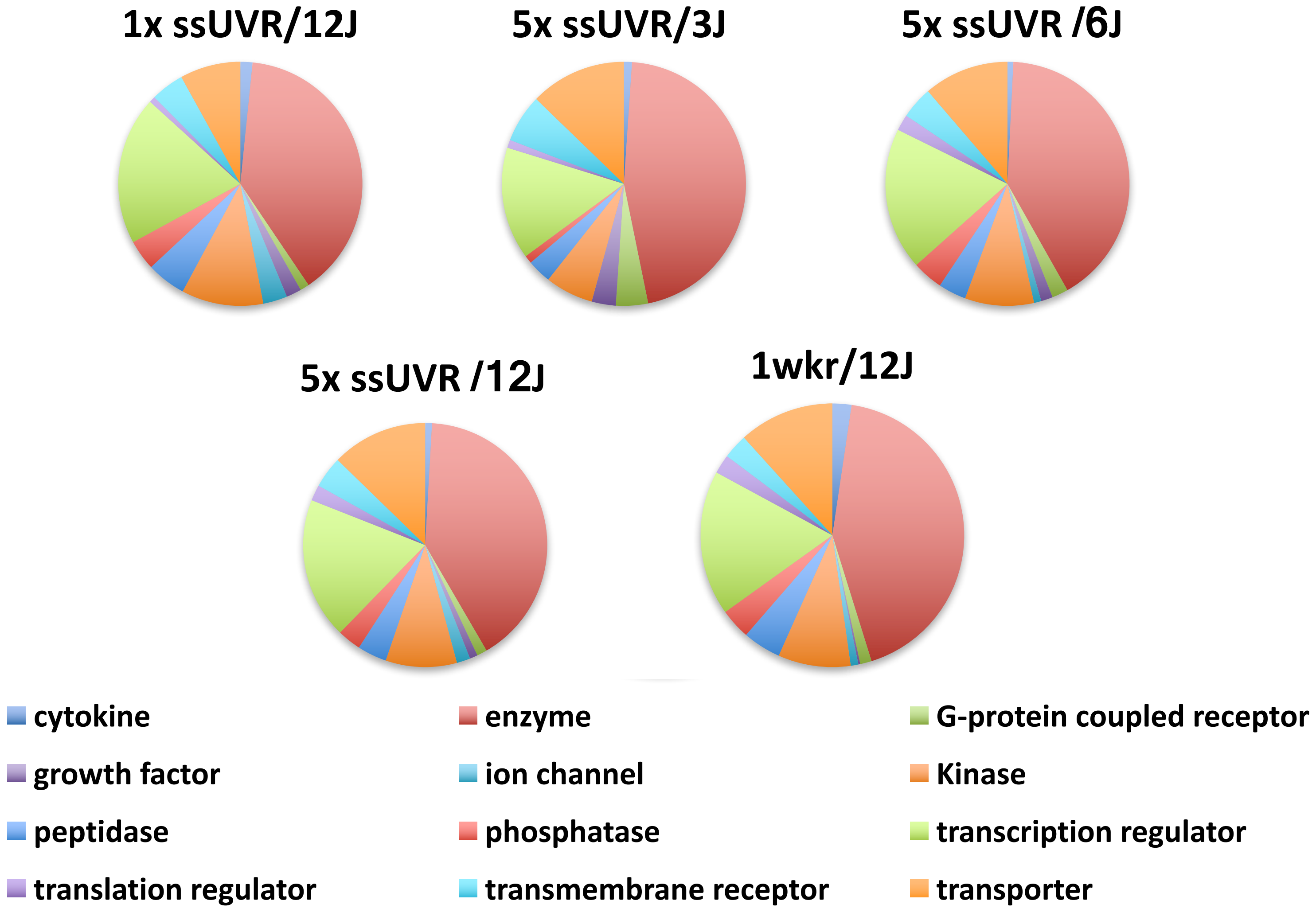

Figure S1. The functional categories of differentially expressed genes in HaCaT cells exposed to ssUVR. The biological function analysis was determined for each gene based on the Ingenuity Pathway Analysis. Number of genes in major categories is shown.

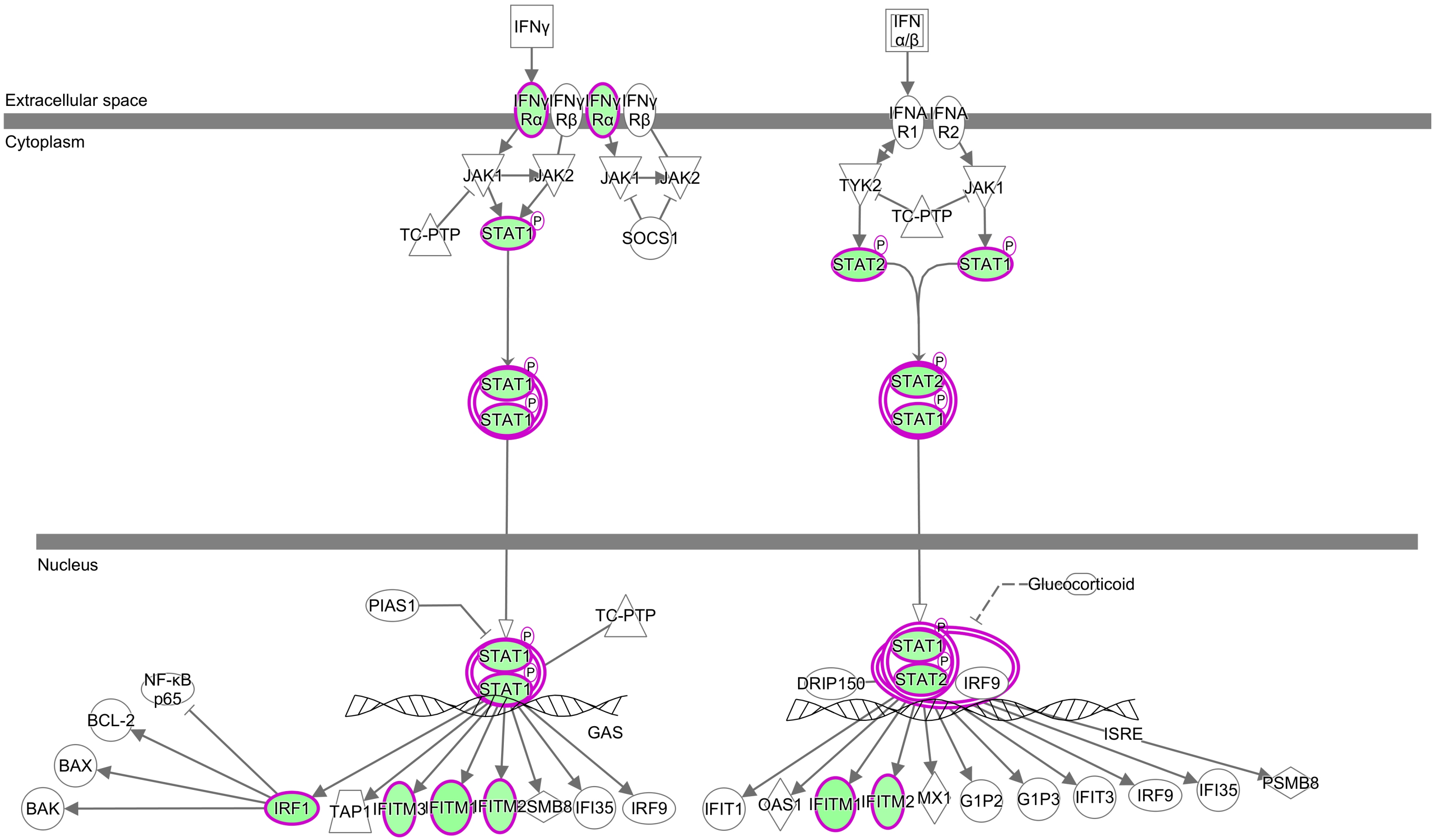

Figure S2. Both type I and II interferon signaling pathway are suppressed in cells exposed to 5 repetitive ssUVR. Purple circle of the node indicates the changed gene expression. Color of each node indicates the regulation of gene expression. Red: up-regulation; Green: doen-regulation.
